# α-Iso-Cubebene Alleviates AMD-like Retinal Injury Through Modulation of Oxidative Stress and Inflammatory Response

**DOI:** 10.3390/cimb48070669

**Published:** 2026-06-29

**Authors:** Ye Ryeong Kim, Ayun Seol, Su Jin Lee, Ji Eun Kim, Hee Jin Song, Su Jeong Lim, Su Ha Wang, Ye Eun Ryu, Young Whan Choi, Sun Il Choi, Dae Youn Hwang

**Affiliations:** 1 Department of Biomaterials Science (BK21 FOUR Program), Laboratory Animals Resources Center, College of Natural Resources and Life Science, Pusan National University, Miryang 50463, Republic of Korea; yr2232@naver.com (Y.R.K.); a990609@naver.com (A.S.); soojl1315@naver.com (S.J.L.); prettyjiunx@naver.com (J.E.K.); hejin1544@naver.com (H.J.S.); nuit4510@naver.com (S.J.L.); dhkdtngk@naver.com (S.H.W.); 5chdtkdlqsl@naver.com (Y.E.R.); 2 Department of Horticultural Bioscience, Pusan National University, Miryang 50463, Republic of Korea; ywchoi@pusan.ac.kr; 3 Research Institute, Graduate School of Cancer Science and Policy, National Cancer Center, Goyang 10408, Republic of Korea; 4 Longevity & Wellbeing Research Center, Life and Industry Convergence Research Institute, Pusan National University, Miryang 50463, Republic of Korea

**Keywords:** α-iso-cubebene, AMD, oxidative stress, inflammation, VEGF

## Abstract

Although oxidative stress plays a critical role in age-related macular degeneration (AMD) progression, natural product–derived single compounds against AMD remain largely unexplored. We investigated the protective effects and underlying mechanism of α-iso-cubebene against AMD-like retinal injury. Alterations in key phenotypes for AMD were analyzed in AMD-mimicking models using ARPE-19 cells co-treated with blue light (BL) and N-retinylidene-N-retinylethanolamine (A2E), as well as BL-exposed BALB/c mice. In BL+A2E-treated ARPE-19 cells, α-iso-cubebene reduced intracellular reactive oxygen species (ROS) and nitric oxide (NO) production and restored superoxide dismutase (SOD) activity and nuclear factor erythroid 2–related factor 2 (Nrf2), suggesting enhancement of the antioxidant defense system. Furthermore, α-iso-cubebene improved cell viability, reduced apoptotic cell populations, and regulated apoptosis-related signaling pathways under oxidative stress conditions. It also attenuated cyclooxygenase-2 (COX-2)-mediated inducible nitric oxide synthase (iNOS) signaling and was associated with reduced inflammasome-related signaling. Importantly, these protective effects were consistently observed regarding the protection of histopathological structure and normalization of inflammatory cytokines in the retina of BL-exposed BALB/c mice. Collectively, our results demonstrate that α-iso-cubebene, as a potential therapeutic candidate, alleviates AMD-like retinal injury and was associated with enhanced antioxidant responses and reduced inflammatory and apoptotic signaling markers.

## 1. Introduction

Age-related macular degeneration (AMD) represents one of the most prevalent retinal degenerative disorders affecting the elderly population, and is a major contributor to vision impairment worldwide [1]. The progression of AMD is characterized by pathological alterations in the macula, including the accumulation of drusen deposits, which are hallmarks of early-stage, or dry, AMD [2]. As the disease advances, dry AMD may progress to neovascular, or wet, AMD, which is marked by the formation of choroidal neovascular membranes and severe central vision loss [2]. This pathological neovascularization is primarily driven by dysregulated vascular endothelial growth factor (VEGF) signaling [3,4]. Consequently, current therapeutic strategies for neovascular AMD mainly rely on intravitreal anti-VEGF injections, often in combination with photodynamic therapy to suppress aberrant vessel formation [5]. Although combined anti-VEGF and photodynamic therapy has improved the clinical management of advanced AMD, its therapeutic benefits are frequently transient, and high recurrence rates within six months continue to present major challenges for long-term visual preservation [6,7,8,9]. Moreover, effective pharmacological treatments for progressive dry AMD remain limited. Apart from surgical implantation of telescopic lenses to enhance visual function, preventive strategies focusing on modifiable risk factors—including smoking cessation, dietary regulation, and cardiovascular health management—remain central to disease control [10]. In this context, daily supplementation with antioxidant nutrients, such as lutein and zeaxanthin, has been shown to slow AMD progression by increasing serum concentrations, enhancing macular pigment optical density, improving contrast sensitivity, and accelerating recovery from photostress [11].

Because oxidative damage is considered a central contributor to retinal degeneration during AMD progression, increasing attention has been directed toward naturally derived antioxidant compounds as potential therapeutic agents [12]. Due to its high oxygen consumption and continuous exposure to light, retinal tissue is especially susceptible to oxidative injury. Additionally, AMD is associated with the accumulation of oxidative stress markers, including lipofuscin deposits, which promote chronic inflammation and pathological angiogenesis [13,14]. Numerous studies have demonstrated that antioxidants can attenuate oxidative injury in retinal cells, thereby contributing to the preservation of visual function [15,16,17]. In addition, antioxidants from natural products offer a multi-targeted therapeutic approach that aligns with the multi-factorial nature of AMD, for which no definitive cure is currently available [12,18]. Based on these characteristics of antioxidants from natural products, single compounds derived from natural products with high antioxidant activities have received a lot of attention as treatments for AMD; however, the number of single compounds whose therapeutic effects against AMD have been evaluated is very limited. Among the compounds isolated from natural products, only a limited number of compounds, including lutein, resveratrol, curcumin, quercetin, and epigallocatechin gallate (EGCG), have shown beneficial protective effects on ARPE-19 cells with AMD phenotypes [19,20,21]. Similar anti-AMD effects have also been detected in AMD model animals after treatment with a few single compounds, such as resveratrol, quercetin, and curcumin [22,23,24]. Therefore, identifying a novel single compound with potential for AMD treatment has important implications because its well-defined chemical structure provides key advantages in drug development by enabling precise structure–activity relationship analysis, target-specific optimization, and improved reproducibility.

Meanwhile, *Schisandra chinensis* is a widely used medicinal herb that exhibits diverse pharmacological activities, including beneficial effects on cardiovascular health [25,26,27]. This plant contains α-iso-cubebene, a dibenzocyclooctadiene lignan with well-documented antioxidant properties, suggesting potential utility in regulating oxidative stress–related pathological processes, such as vascular smooth muscle cell proliferation [28]. Also, this compound has been reported to exert anti-inflammatory and immunomodulatory effects, including the suppression of amyloid β–induced neuroinflammation, protection against 6-hydroxydopamine-induced microglial cell death [29,30], and attenuation of lung inflammation and lipopolysaccharide-induced splenocyte apoptosis [31]. In addition, this compound inhibits tumor necrosis factor-α (TNF-α)-stimulated endothelial–monocyte adhesion by reducing ROS generation [32]. Nevertheless, whether α-iso-cubebene exerts protective effects against AMD-associated retinal degeneration and the molecular pathways involved remain largely unknown.

In the present study, we identified α-iso-cubebene—a novel single compound derived from natural products with anti-AMD-like retinal injury effects—through the evaluation of AMD phenotypes in N-retinylidene-N-retinylethanolamine (A2E)-laden ARPE-19 cells and BALB/c mice subjected to blue light (BL) exposure. Furthermore, we explored the molecular mechanisms underlying its antioxidant, anti-inflammatory, and anti-angiogenic activities.

## 2. Materials and Methods

### 2.1. Preparation of α-Iso-Cubebene

**Figure 1:**
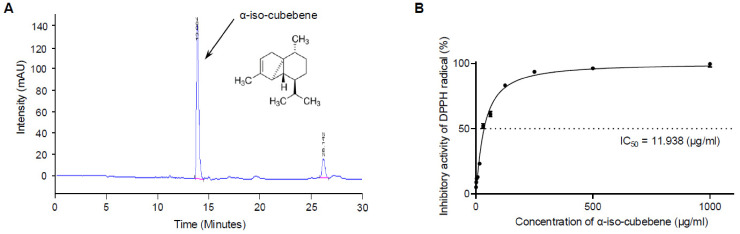
Isolation and antioxidative activity of α-iso-cubebene: (**A**) HPLC profile of α-iso-cubebene. The active fraction contained almost pure (>99%) α-iso-cubebene, as verified by HPLC analysis at 254 nm; (**B**) DPPH radical-scavenging activity of α-iso-cubebene. Results are reported as the mean ± SD. Abbreviations: DPPH, 2,2-diphenyl-1-picrylhydrazyl; IC_50_, half-maximal inhibitory concentration.

α-Iso-cubebene was isolated and purified from the dried fruit of *Schisandra chinensis* as described previously [32]. Briefly, the fruits of *Schisandra chinensis* were collected in September 2005 from Mungyeong, Gyeongbuk, Republic of Korea. A voucher specimen (Accession number: SC-PDRL-1) was deposited in the Herbarium of Pusan National University (Busan, Republic of Korea). The ground powder of these dried fruits (2.5 kg) was extracted at room temperature with hexane, CHCl_3_, and MeOH. The hexane extract (308 g) was evaporated in a vacuum centrifuge and separated on a silica gel column (40 μm, 100 × 10 cm, Baker, NJ, USA) using a step gradient of 0%, 5%, and 20% EtOAc in hexane and 5% MeOH in CHCl_3_ to obtain 38 fractions. Fraction 1 (KH1PA, 3689 mg) was further separated on a silica gel column (100 × 3.0 cm) using CH_2_Cl_2_ to yield 9 subfractions. Next, subfraction 2 (KH1PAIB, 999 mg) was separated on a silica gel column (100 × 3.0 cm) using 0.5% acetone in CH_2_Cl_2_ to yield α-iso-cubebene (316 mg). Chemical purities were verified by high-performance liquid chromatography using a Phenomenex Luna C18 column (Phenomenex, 150 × 4.6 mm ID, 5 μm particle size) and an acetonitrile–water gradient at a flow rate of 1.0 mL per min. The chemical structure of α-iso-cubebene was then verified by liquid chromatography–mass spectrometry (LC-MS) using a Bruker BioApex FT mass spectrometer (Bruker Daltonics, Billerica, MA, USA) and nuclear magnetic resonance (NMR) spectroscopy using a Varian Inova 500 spectrometer (Varian, Palo Alto, CA, USA) (Figure 1A).

### 2.2. Scavenging Activity of α-Iso-Cubebene Against DPPH Radicals

The free radical–scavenging activity of α-iso-cubebene was determined as previously described [33,34,35]. Briefly, a dry powder sample was dissolved in 50% EtOH (100 µL) to obtain 12 different concentrations (1 to 2000 µg/mL), which were then mixed with 100 µL of 0.1 mM 2,2-diphenyl-1-picrylhydrazyl (DPPH, Sigma-Aldrich Co., St. Louis, MO, USA) in a 95% ethanol solution, or with 100 µL of 95% ethanol solution as a control. The mixtures were incubated at room temperature for 30 min, and then the absorbance at 517 nm was measured using a Versa Max plate reader (Molecular Devices, Sunnyvale, CA, USA). The DPPH-derived radical-scavenging activity of each α-iso-cubebene sample was measured as the percent decrease in absorbance relative to the control for the calculation of the IC_50_ value.

### 2.3. Cell Culture

The human retinal pigment epithelial (RPE) cell line (ARPE-19) was purchased from the American Type Culture Collection (ATCC, Manassas, VA, USA) and cultured in Dulbecco’s Modified Eagle’s Medium (DMEM, Welgene, Gyeongsan-si, Republic of Korea) supplemented with 10% fetal bovine serum (FBS), 2 mM glutamine, 100 U/mL penicillin, and 100 μg/mL streptomycin. Cells were grown in a humidified atmosphere containing 5% CO_2_ and 95% air at 37 °C.

### 2.4. Treatment of BL+A2E-Treated ARPE-19 Cells with α-Iso-Cubebene

An AMD model induced by BL and A2E co-treatment was established following a previously published method [35]. Briefly, ARPE-19 cells were seeded at a density of 3 × 10^4^ cells/well in 6-well plates and randomly divided into the following two groups: non-treated group (NT group) and A2E+BL-treated group. The latter group was further classified into five different groups: DMSO-treated group (Veh+A2E+BL-treated group); 100 μM Vitamin C–treated group (VitC+A2E+BL-treated group); 40 μM α-iso-cubebene-treated group (40+A2E+BL-treated group); 80 μM α-iso-cubebene-treated group (80+A2E+BL-treated group); and 160 μM α-iso-cubebene-treated group (160+A2E+BL-treated group). Vitamin C was used as a positive control because of its well-established antioxidant activity [15,16]. When ARPE-19 cells reached 70–80% confluence, they were treated with α-iso-cubebene (40, 80, or 160 μM), equal-volume DMSO, or Vitamin C for 24 h, and then treated with 20 μM A2E for 24 h. Subsequently, the cells were exposed to BL (450 nm, 2000 lux) (SL-S2500, S tech LED, Goyang-si, Gyeonggi-do, Republic of Korea) for 10 min and allowed to recover for 24 h. Meanwhile, the optimal concentration of A2E was determined based on previous studies [35] and the results of our preliminary data (Supplementary Figure S1). Also, the optimal dosage of α-iso-cubebene was selected based on non-cytotoxic concentrations (Supplementary Figure S2).

### 2.5. Cell Viability Assay

To determine the optimal concentration of α-iso-cubebene for experiments (i.e., the concentration with the highest protective efficacy without inherent cytotoxicity), ARPE-19 cells were seeded at ~3 × 10^4^ cells/well in 96-well plates, allowed to reach 70–80% confluence, then treated with 40, 80, or 160 μM α-iso-cubebene for 24 h. The medium was discarded and replaced with 200 μL of fresh DMEM plus 50 μL of MTT solution (20 mg/mL in 1 × PBS), followed by incubation at 37 °C for 4 h. The formazan precipitate produced from MTT by viable cells was dissolved in DMSO (Duchefa Biochemie, Haarlem, The Netherlands), and the absorbance of each well was measured at 570 nm using a Versa Max plate reader (Molecular Devices) as an estimate of the number of viable cells remaining.

### 2.6. Apoptosis Assay

The distribution of apoptotic cells was analyzed using a MuseTM Annexin V and Dead Cell Kit (Millipore Co., Billerica, MA, USA), according to the manufacturer’s protocol. Briefly, ARPE-19 cells treated with the indicated concentrations were harvested and resuspended in MuseTM Annexin V and Dead Cell Kit reaction reagent for 20 min at room temperature. Staining was then analyzed using a MuseTM Cell Analyzer (Millipore). Cells were classified into four populations: live cells [Annexin V (−) and 7-AAD (−)], early apoptotic cells [Annexin V (+), 7-AAD (−)], late apoptotic cells [Annexin V (+), 7-AAD (+)], and mostly nuclear debris [Annexin V (−), 7-AAD (+)].

### 2.7. Measurement of Intracellular ROS

The levels of intracellular reactive oxygen species (ROS) were measured in each treatment group by staining with 2′,7′-dichlorofluorescein diacetate (DCF-DA, Sigma-Aldrich Co.), a cell-permeable compound that is non-fluorescent in the reduced state (DCFH). Cells treated with the indicated concentrations were incubated with 10 µM DCF-DA for 30 min at 37 °C and washed twice with 1 × PBS. The green fluorescence resulting from ROS-mediated oxidation was visualized under a fluorescence microscope (EVOS M5000, Thermo Fisher Scientific Inc., Wilmington, DE, USA) at 200× magnification.

### 2.8. Measurement of NO Production

The production of nitric oxide (NO) by A2E and BL co-treatment was estimated by measuring the medium accumulation of nitrite—which is a stable oxidation product of NO generated in the presence of oxygen—using the Griess reaction. Briefly, ARPE-19 cells were treated with the indicated concentrations, and the medium was collected and mixed 1:1 (100 µL of each) with Griess reagent (Invitrogen, Waltham, MA, USA) in 96-well plates. The absorbance of each well was measured at 540 nm using a Versa Max plate reader (Molecular Devices). A standard curve was constructed using known concentrations of sodium nitrite to quantify sample nitrite concentrations.

### 2.9. Analysis of SOD Activity

Superoxide dismutase (SOD) activity in ARPE-19 cells was assessed using a colorimetric SOD assay kit (Dojindo Molecular Technologies Inc., Rockville, MD, USA). Briefly, ARPE-19 cells treated with the indicated concentrations were lysed by repetitive freezing and thawing in 100 μL of 1 × PBS. The lysates were collected by centrifugation at 5000× *g* for 5 min and diluted 1/1, 1/2, 1/2^2^, 1/2^3^, 1/2^4^, 1/2^5^, and 1/2^6^ with 1 × PBS. Aliquots of lysate sample (20 μL) were then transferred to the individual wells of 96-well plates and mixed with 200 μL of WST-1 working solution and 20 μL of enzyme working solution. The enzyme reaction was induced by incubation at 37 °C for 20 min, after which well absorbance was measured at 450 nm using a spectrophotometer. Finally, SOD activity was calculated as

SOD activity (inhibition rate %) = [(A blank 1 − A blank 3) − (A sample − A blank 2)]/(A blank 1 − A blank 3) × 100,

where A blanks 1, 2, and 3 indicate the absorbances of blanks 1, 2, and 3, respectively, and “A sample” is the sample absorbance.

### 2.10. Western Blotting Analysis

Total proteins were obtained from the ARPE-19 cells of each group using the Pro-Prep Protein Extraction Solution (Intron Biotechnology Inc., Seongnam, Republic of Korea), according to the manufacturer’s protocol. The concentrations of total proteins were determined using a Pierce™ BCA Protein Assay Kit (Thermo Fisher Scientific). An appropriate amount of protein (20 μg) was separated by 4–20% sodium dodecyl sulfate–polyacrylamide gel electrophoresis (SDS-PAGE) for 2 h, and transferred to nitrocellulose membranes at 40 V for 2 h. Membranes were then incubated overnight at 4 °C with the specific primary antibodies (Supplementary Table S1). Membranes were then immersed in washing buffer (137 mM NaCl, 2.7 mM KCl, 10 mM Na_2_HPO_4_, and 0.05% Tween 20) and incubated in horseradish peroxidase (HRP)-conjugated goat anti-rabbit IgG (1:2000, Invitrogen) at room temperature for 1 h. Blots were developed using Amersham ECL Select Western blotting detection reagent (GE Healthcare, Little Chalfont, UK). Chemiluminescence signals from specific bands were detected using the FluorChem FC2 Imaging System (Alpha Innotech Co., San Leandro, CA, USA).

### 2.11. RT-qPCR Analysis

To conduct quantitative real-time PCR (RT-qPCR) analysis, total RNA was purified from ARPE-19 cells and eye tissues using RNAzol (Tet-Test Inc., Friendswood, TX, USA). After determination of total RNA concentration and purity by spectrophotometry, complementary DNA (cDNA) was synthesized using Invitrogen Superscript II reverse transcriptase (Thermo Fisher Scientific Inc.). Quantitative PCR was performed using 2 × Power SYBR Green (6 μL, Toyobo Life Science, Osaka, Japan) and the primer pairs (Supplementary Table S2). The thermal cycling conditions for RT-qPCR were 40 cycles of denaturation at 95 °C for 15 s, annealing at 70 °C for 60 s, and extension at 70 °C for 60 s. Each reaction included 1 μL of cDNA template. Fluorescence intensities were measured at the end of the extension phase of each cycle. Threshold values for sample fluorescence intensities were set manually, and reaction cycles at which PCR products exceeded these thresholds during the exponential phase were considered threshold cycles (Ct). The expression levels of *TNF-α*, *IL-6*, *IL-1β*, and *NF-κB* genes were quantified relative to that of the housekeeping gene encoding β-actin based on a comparison of the Ct values at a constant fluorescence intensity [36].

### 2.12. Experimental Design for Animal Study

The protocol was reviewed and approved by the Pusan National University (PNU) Institutional Animal Care and Use Committee (IACUC) for the therapeutic effects of α-iso-cubebene in an AMD animal model (Approval no. PNU-2026-0773). All mice were housed at the PNU Laboratory Animal Resources Center (LARC), accredited by the Republic of Korea Food and Drug Administration (KFDA) (unit 000231) and the Association for Assessment and Accreditation of Laboratory Animal Care International (AAALAC International) (unit 001525). Male BALB/c mice (7-weeks-old) originated from Taconic Co. (Germantown, NY, USA), and were purchased from Samtako BioKorea Co., Ltd. (Osan, Republic of Korea). The mice were bred under specific pathogen-free conditions (SPF) (50 ± 10% relative humidity and 23 ± 2 °C temperature) under a strict light/dark cycle.

Briefly, the seven-week-old BALB/c mice (male, *n* = 21) were assigned to either non-exposed mice (NT group, *n* = 7) or BL-exposed mice (*n* = 14, BL-exposed group). The latter group was subdivided into the Vehicle (1 × PBS)-injected mice (Vehicle+BL-treated group, *n* = 7) and the α-iso-cubebene-injected mice (100 μM, α-iso-cubebene+BL-treated group, *n* = 7). After adapting to the dark cages for seven days, all mice in the BL-exposed groups were injected intravitreally with 100 μM of α-iso-cubebene (1 μL) or the same volume of 1 × PBS once (1 μL) under the dark condition. The dose of α-iso-cubebene (100 μM) and the evaluation period for 24 h were selected based on previous studies employing acute BL-induced retinal injury models and intravitreal administration of antioxidant compounds [37,38]. As the primary objective of this study was to provide proof-of-concept evidence for the protective effects of α-iso-cubebene against AMD-like retinal injury, a single-dose design with short-term evaluation was adopted. Nevertheless, we acknowledge that additional studies investigating dose–response relationships and long-term therapeutic effects are required to further verify its efficacy and molecular mechanism. Furthermore, these mice were then exposed to BL (430 nm, 600 lux) for 2 h and allowed to rest for 24 h in the dark. Finally, all mice were euthanized using CO_2_ inhalation with a minimum purity of 99.0% based on the *American Veterinary Medical Association (AVMA) Guidelines for the Euthanasia of Animals*. The death of the mice was confirmed by ascertaining cardiac and respiratory arrest, dilated pupils, and the absence of reflexes. The eye tissues were then collected from euthanized BALB/c mice and subjected to histological and RT-qPCR analyses.

### 2.13. Histological Analyses

The histopathological analyses of eye tissue were conducted as described in a previous study [39]. Briefly, the eye tissues collected from the BALB/c mice were fixed in 10% neutral-buffered formaldehyde (pH = 6.8), dehydrated in an alcohol dilution series (70 to 100%), and embedded in paraffin wax. These tissues were sectioned into 4 µm thick slices, and subsequently deparaffinized and rehydrated using xylene solution (DaeJung Chemicals, Siheung, Republic of Korea) and an alcohol dilution series (100 to 70%). After removing the residues with distilled water, the tissue sections were stained with hematoxylin and eosin (H&E, Sigma-Aldrich Co.), and the histopathological changes in the eye tissues were analyzed using the Leica Application Suite (LAS, Leica Microsystems). The thicknesses of the whole retina, outer nuclear layer (ONL), and inner nuclear layer (INL) in the mounted eye tissue were measured using the Leica Application Suite (Leica Microsystems, Heerbrugg, Switzerland). During thickness analyses, three different points, including the thickest point, thinnest point, and intermediate point, were measured, and the mean value was calculated to minimize variation caused by surface irregularities in each layer. Also, the thickness analyses were performed by researchers blinded to the injection groups to minimize observer bias.

### 2.14. Statistical Analyses

All data are expressed as the mean ± standard deviation (SD) of at least three technical replicates. All statistical analyses were performed using GraphPad Prism 8.0 (GraphPad Software Inc., San Diego, CA, USA). Group means were compared using one-way analysis of variance (ANOVA) followed by Tukey’s post hoc test for multiple comparisons. Additionally, a correlogram was generated using the corrplot package (Version 4.3.3) in R software environment (R Core Team, 2026). Correlations between the concentration of α-iso-cubebene and other parameters in ARPE-19 cells were analyzed using Pearson’s correlation coefficient in R, and the results are represented as scatterplots. A value of *p* < 0.05 was considered statistically significant. Individual *p* values are provided in the figure legends.

## 3. Results

### 3.1. Antioxidant Activity of α-Iso-Cubebene

We first evaluated the antioxidant activity of isolated α-iso-cubebene to evaluate its potential for AMD-like retinal injury treatment because the induction and progression of AMD are closely related to oxidative stress. To achieve this, the scavenging activity against DPPH was analyzed at different concentrations of α-iso-cubebene. The DPPH radical-scavenging activity increased rapidly in a dose-dependent manner at a range of concentrations from 1 to 1000 µg/mL. The IC_50_ value of α-iso-cubebene was determined at 11.938 µg/mL (Figure 1B). Therefore, these findings suggest that α-iso-cubebene possesses potent antioxidant activity and may have therapeutic potential for oxidative stress–related damage, such as AMD-like retinal injury.

### 3.2. Protective Effects of α-Iso-Cubebene on the Antioxidant Defense System of BL+A2E-Treated ARPE-19 Cells

**Figure 2:**
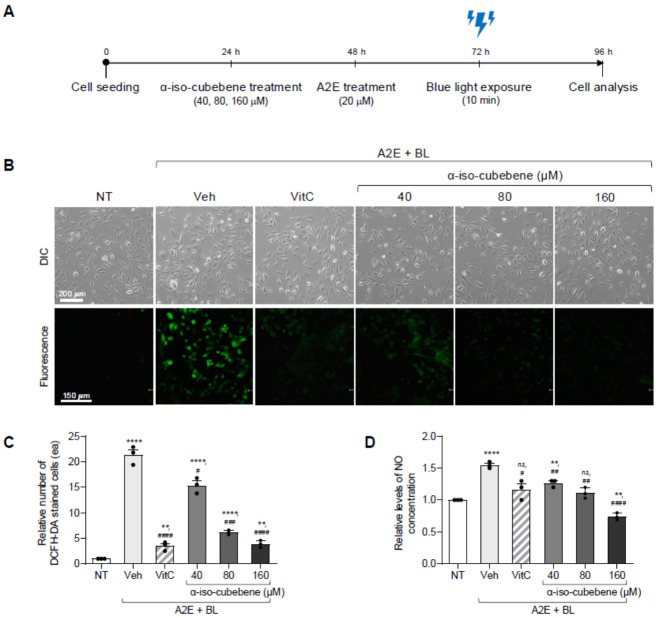
Concentration of ROS and NO in α-iso-cubebene+A2E+BL-treated ARPE-19 cells: (**A**) Experimental schedule for ARPE-19 cells; (**B**) Fluorescence image of DCFH-DA-stained cells; (**C**) Level of intracellular ROS production in ARPE-19 cells. Cells were loaded with the ROS-sensitive fluorescent dye DCFH-DA, and green fluorescent emission was measured using a fluorescence microscope (200× magnification); (**D**) Level of NO released from ARPE-19 cells using the Griess reaction. Results are presented as the mean ± SD of three independent experiments. **** *p* < 0.0001, ** *p* < 0.01, and versus the NT group; ^####^ *p* < 0.0001, ^###^ *p* < 0.001, ^##^ *p* < 0.01, and ^#^ *p* < 0.05 versus the Vehicle+A2E+BL-treated group; ns: not significant. Abbreviations: NT, non-treated; Veh, vehicle (DMSO); VitC, Vitamin C; DCFH-DA, 2′,7′-dichlorodihydrofluorescein diacetate; A2E, N-retinylidene-N-retinylethanolamine; BL, blue light; DIC, differential interference contrast; NO, nitric oxide.

**Figure 3:**
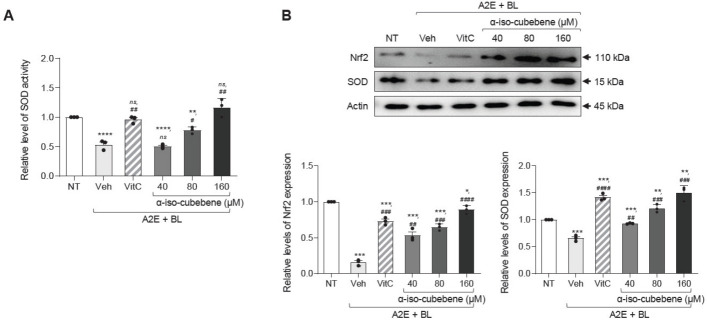
Levels of antioxidant parameters in α-iso-cubebene+A2E+BL-treated ARPE-19 cells: (**A**) SOD activity; (**B**) Expression levels of SOD and Nrf2 proteins. Results are the mean ± SD of three independent experiments. **** *p* < 0.0001, *** *p* < 0.001, ** *p* < 0.01, and * *p* < 0.05 versus the NT group; ^####^ *p* < 0.0001, ^###^ *p* < 0.001, ^##^ *p* < 0.01, and ^#^ *p* < 0.05 versus the Vehicle+A2E+BL-treated group. Abbreviations: NT, non-treated; Veh, vehicle (DMSO); VitC, Vitamin C, as a positive control; BL, blue light; A2E, N-retinylidene-N-retinylethanolamine; SOD, superoxide dismutase; Nrf2, nuclear factor erythroid 2–related factor 2.

BL+A2E-treated ARPE-19 cells have been reported to recapitulate core characteristics of dry AMD featuring lipofuscin accumulation, including the generation of ROS and stimulation of NO synthesis, which ultimately induces a state of oxidative stress and activates apoptotic and nuclear factor kappa B (NF-*κ*B)-dependent inflammatory pathways [40,41]. Therefore, we investigated whether α-iso-cubebene could prevent alterations in the antioxidant defense system induced by AMD-related stress. To achieve this, alterations in intracellular ROS and NO levels, SOD activity and expression, and Nrf2 expression were analyzed in BL+A2E-treated ARPE-19 cells after pretreatment with α-iso-cubebene (Figure 2A). The number of DCFH-DA-stained cells was higher in the Vehicle+BL+A2E-treated group than in the NT group. However, this significantly decreased in a dose-dependent manner after treatment with α-iso-cubebene, although the highest reduction was observed in the 160 μM α-iso-cubebene-treated group (Figure 2B,C). A similar dose-dependent reduction was observed in NO concentration (18.10%, 28.23%, and 52.16%) (Figure 2D). Conversely, the SOD activity and the expression levels of SOD and Nrf2 significantly increased following α-iso-cubebene treatment, showing the opposite trend to ROS and NO levels (Figure 3A,B). Taken together, these results indicate that α-iso-cubebene mitigates A2E/BL-induced oxidative stress, at least in part, by enhancing endogenous antioxidant defense mechanisms.

### 3.3. Protective Effects of α-Iso-Cubebene on the Apoptosis of BL+A2E-Treated ARPE-19 Cells

**Figure 4:**
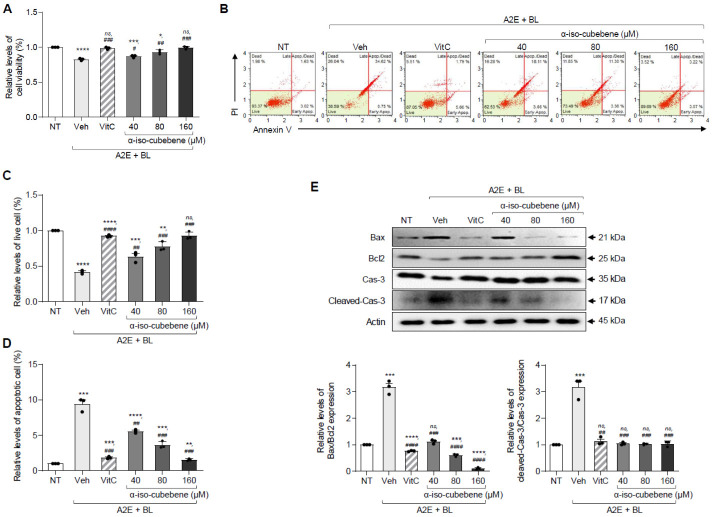
Levels of apoptotic parameters in α-iso-cubebene+A2E+BL-treated ARPE-19 cells: (**A**) Levels of cell viability. Three microplate wells were established per treatment group, and the optical density of formazan produced from MTT by viable cells was measured in triplicate; (**B**) Flow cytometric analysis for Annexin V and 7-AAD. Red lines indicate the gating boundaries used for flow cytometric analysis; (**C**) Number of apoptotic cells; (**D**) Number of live cells. Initial cell population gating was based on the cell size vs. Annexin V. Obvious debris was gated out from the total cell population. Three wells per treatment group were prepared for apoptosis staining, and the numbers of dead and live cells were measured in triplicate; (**E**) Expression levels of pro- and anti-apoptotic proteins. Three plates per treatment group were used to prepare cell homogenates, and Western blotting was conducted in triplicate for each sample. Results are presented as the mean ± SD of three independent experiments. **** *p* < 0.0001, *** *p* < 0.001, ** *p* < 0.01, and * *p* < 0.05 versus the NT group; ^####^ *p* < 0.0001, ^###^ *p* < 0.001, ^##^ *p* < 0.01, and ^#^ *p* < 0.05 versus the Vehicle+A2E+BL-treated group; ns: not significant. Abbreviations: NT, non-treated; Veh, vehicle; VitC, Vitamin C; MTT, 3-(4,5-dimethylthiazol-2-yl)-2,5-diphenyltetrazolium bromide; 7-AAD, 7-aminoactinomycin D; Cas-3, caspase-3; Bax, Bcl-2-associated X protein; Bcl2, B-cell lymphoma 2; PI, propidium iodide; A2E, N-retinylidene-N-retinylethanolamine; BL, blue light.

To investigate whether α-iso-cubebene can protect against AMD-related cell death, alterations in cell viability, number of apoptotic cells, and expression of apoptotic proteins were analyzed in BL+A2E-treated ARPE-19 cells after pretreatment with α-iso-cubebene. Consistent with the protective effects of α-iso-cubebene on antioxidant defense systems, pretreatment with α-iso-cubebene dose-dependently enhanced ARPE-19 cell viability following A2E plus BL co-treatment, as measured by the MTT assay (Figure 4A). Also, Annexin V and PI staining revealed a 9.4-fold increase in the number of apoptotic cells following Vehicle+A2E+BL treatment compared to the non-treated group, which was significantly reversed by all tested concentrations of α-iso-cubebene (40, 80, and 160 μM) (Figure 4B–D). However, the number of apoptotic cells remarkably decreased after treatment with α-iso-cubebene, while the number of live cells increased (Figure 4C,D). In particular, the numbers of apoptotic and live cells in the 160 μM α-iso-cubebene+A2E+BL group were nearly restored to those in the NT group (Figure 4C,D). Furthermore, the recovery effects of α-iso-cubebene in apoptotic cell numbers were completely reflected in the expression levels of the apoptotic proteins. The relative level of Bax/Bcl-2 expression was higher in the Vehicle+A2E+BL-treated group than in the non-treated group. However, these levels significantly decreased after pretreatment with α-iso-cubebene (Figure 4E). In addition, the Vehicle+A2E+BL-treated group also exhibited significantly elevated levels of cleaved Cas-3/Cas-3, which were also reversed by all tested doses of α-iso-cubebene (Figure 4E). These observations suggest that the cytoprotective effect of α-iso-cubebene is associated with inhibition of Cas-3-mediated apoptotic signaling.

### 3.4. Protective Effects of α-Iso-Cubebene on the Angiogenesis of BL+A2E-Treated ARPE-19 Cells

**Figure 5:**
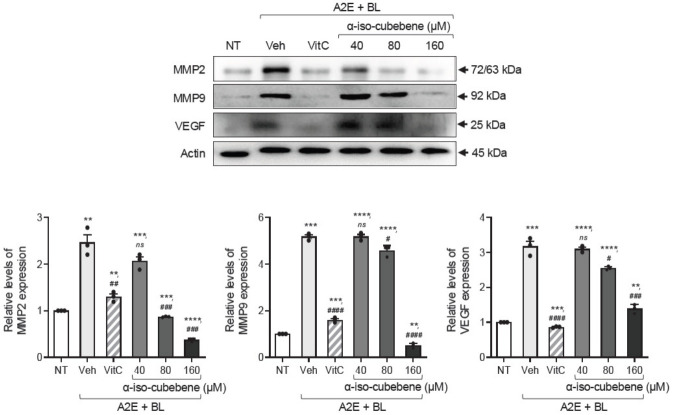
Expression levels of pro-angiogenic proteins in α-iso-cubebene+A2E+BL-treated ARPE-19 cells. Three plates per treatment group were used to prepare total protein homogenates, and target protein expression levels were estimated by Western blotting. Results are presented as the mean ± SD of three independent experiments. **** *p* < 0.0001, *** *p* < 0.001, and ** *p* < 0.01 versus the NT group; ^####^ *p* < 0.0001, ^###^ *p* < 0.001, ^##^ *p* < 0.01, and ^#^ *p* < 0.05 versus the Vehicle+A2E+BL-treated group; ns: not significant. Abbreviations: NT, non-treated; Veh, vehicle; VitC, Vitamin C; MMP, matrix metalloproteinase; BL, blue light; A2E, N-retinylidene-N-retinylethanolamine; VEGF, vascular endothelial growth factor.

To examine whether α-iso-cubebene can suppress AMD-related angiogenesis, alterations in the expression levels of the pro-angiogenic proteins VEGF and matrix metalloproteinases (MMP2 and MMP9) were analyzed in BL+A2E-treated ARPE-19 cells after pretreatment with α-iso-cubebene. Overall, all three proteins exhibited similar expression patterns across the experimental groups. The expression levels of these proteins increased in the Vehicle+A2E+BL-treated group compared to the NT group, while α-iso-cubebene dose-dependently reversed the upregulation of MMP2 at all tested doses, and dose-dependently reversed both MMP9 and VEGF upregulation at 80 and 160 μM compared to the Vehicle+A2E+BL-treated group (Figure 5). These findings provide indirect evidence that α-iso-cubebene can suppress the angiogenic signaling induced by A2E and BL co-treatment in ARPE-19 cells.

### 3.5. Protective Effects of α-Iso-Cubebene on the Inflammation of BL+A2E-Treated ARPE-19 Cells

**Figure 6:**
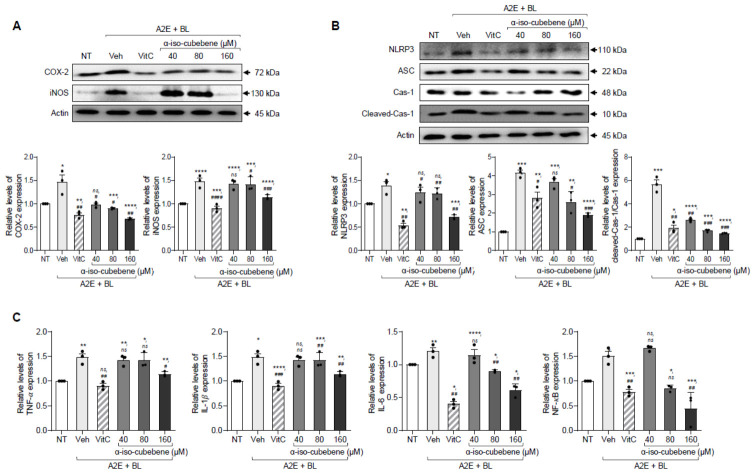
Levels of inflammation-related parameters in α-iso-cubebene+A2E+BL-treated ARPE-19 cells: (**A**) Expression levels of COX-2 and iNOS proteins; (**B**) Expression levels of inflammasome proteins. Three plates per treatment group were used to prepare total cell lysates, and Western blotting was used to estimate protein expression levels. Each experiment was performed in triplicate; (**C**) Transcriptional levels of inflammatory cytokine genes. Three wells per treatment group were used to prepare the total RNA samples, and RT-qPCR was performed in triplicate for each sample. Results are presented as the mean ± SD of three independent experiments. **** *p* < 0.0001, *** *p* < 0.001, ** *p* < 0.01, and * *p* < 0.05 versus the NT group; ^####^ *p* < 0.0001, ^###^ *p* < 0.001, ^##^ *p* < 0.01, and ^#^ *p* < 0.05 versus the Vehicle+A2E+BL-treated group; ns: not significant. Abbreviations: NT, non-treated; Veh, vehicle; VitC, Vitamin C; COX-2, cyclooxygenase-2; iNOS, inducible nitric oxide synthase; BL, blue light; A2E, N-retinylidene-N-retinylethanolamine; NLRP3, NLR family pyrin domain containing 3; ASC, apoptosis-associated speck-like protein containing a CARD; Cas-1, caspase-1; TNF-*α*, tumor necrosis factor-*α*; IL-6, interleukin 6; IL-1*β*, interleukin 1*β*; NF-*κ*B, nuclear factor kappa-light-chain-enhancer of activated B cells.

Next, we investigated whether α-iso-cubebene can suppress the inflammatory response caused by AMD. To achieve this, alterations in the levels of key parameters for the COX-2-mediated iNOS induction pathway, inflammasome, and inflammatory cytokines were analyzed in BL+A2E-treated ARPE-19 cells after pretreatment with α-iso-cubebene. The expression levels of iNOS and COX-2 were higher in the Vehicle+A2E+BL-treated group than in the NT group. However, the expression levels of COX-2 were dose-dependently reduced across all tested α-iso-cubebene concentrations, while that of iNOS was also significantly reduced in 80 and 160 μM α-iso-cubebene-treated groups (Figure 6A).

The suppressive effects of α-iso-cubebene on iNOS and COX-2 were paralleled by the downregulation of inflammasome components, including NLR family pyrin domain containing 3 (NLRP3), apoptosis-associated speck-like protein containing a CARD (ASC), and cleaved Cas-1 [42]. The expression levels of all three inflammasome proteins were higher in the Vehicle+A2E+BL-treated group than in the NT group. However, these levels decreased in a dose-dependent manner in all α-iso-cubebene+A2E+BL-treated groups compared to the Vehicle+A2E+BL-treated group, although their rates of decline varied (Figure 6B).

Furthermore, the suppression of the iNOS-induced COX-2 pathway and inflammasome activation by α-iso-cubebene was paralleled by reductions in the expression levels of the pro-inflammatory cytokines *TNF-α*, *IL-6*, and *IL-1β*, as well as the reduced expression of NF-κB, the core transcription factor controlling the inflammatory response [43]. The mRNA expression levels of three pro-inflammatory cytokines and NF-κB were significantly lower in α-iso-cubebene+A2E+BL-treated groups compared to the Vehicle+A2E+BL-treated group (Figure 6C). Collectively, α-iso-cubebene attenuated inflammatory signaling induced by A2E and BL exposure, which was accompanied by suppression of the COX-2/iNOS axis and NLRP3 inflammasome activation.

### 3.6. Protective Effects of α-Iso-Cubebene on the Retinal Histopathology and Inflammatory Cytokines of BL-Exposed BALB/c Mice

**Figure 7:**
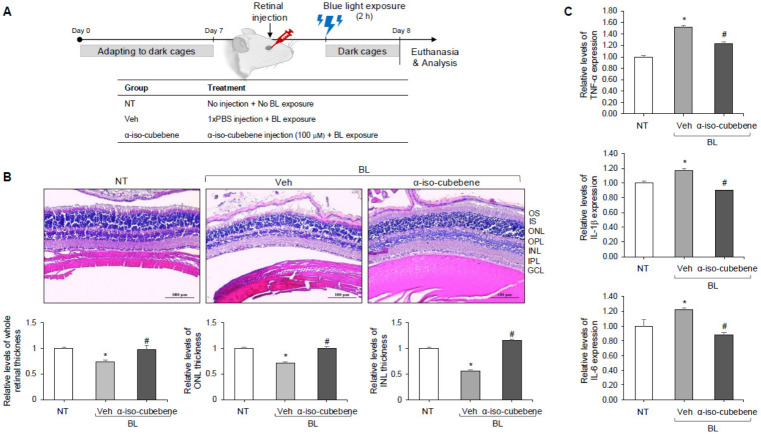
Retinal histopathology and inflammatory cytokine mRNA expression in α-iso-cubebene+BL-treated BALB/c mice: (**A**) Experimental schedule for BALB/c mice; (**B**) Representative H&E-stained retinal sections. The preparation of stained eye sections was performed using three to five mice per group, and measurements of thickness were conducted twice per section; (**C**) Transcriptional levels of inflammatory cytokine genes. The preparation of total RNA samples was performed using two to three mice per group, and measurements of mRNA levels were conducted twice per sample. Data are expressed as the mean ± SD. * *p* < 0.05 vs. the NT group; ^#^ *p* < 0.05 vs. the Veh-injected group. Abbreviations: NT, non-treated; Veh, vehicle; OS, outer segments; IS, inner segments; ONL, outer nuclear layer; OPL, outer plexiform layer; INL, inner nuclear layer; IPL, Inner plexiform layer; GCL, ganglion cell layer; TNF-*α*, tumor necrosis factor-*α*; IL-6, interleukin-6; IL-1*β*, interleukin-1*β*.

To validate the protective effects of α-iso-cubebene on AMD-like retinal injury observed in BL+A2E-treated ARPE-19 cells in an animal model, alterations in the retinal histopathological structure and inflammatory responses were analyzed in BL-exposed BALB/c mice following the retinal injection of α-iso-cubebene (Figure 7A). Histopathological analysis revealed marked thinning of the whole retina and individual retinal layers, including the ONL and INL, in the Vehicle+BL-treated BALB/c mice compared with the non-treated group. α-iso-cubebene injection significantly restored retinal thickness across these layers relative to the Vehicle+BL-treated group (Figure 7B). Also, α-iso-cubebene injection significantly decreased the expression of pro-inflammatory cytokine genes, including TNF-α, IL-1β, and IL-6 (Figure 7C). These findings indicate that α-iso-cubebene can protect retinal structural degeneration and inflammatory responses in retinal tissues under AMD-like pathophysiological conditions in an animal model. In addition, our findings suggest that the protective effects of α-iso-cubebene in ARPE-19 cells are successfully reproduced in the retina of BL-exposed BALB/c mice.

### 3.7. Correlation Between α-Iso-Cubebene Concentration and AMD-Related Phenotypes

Finally, we analyzed the correlation between the α-iso-cubebene concentration and AMD phenotypes from results obtained in ARPE-19 cells to evaluate the association between α-iso-cubebene concentration and AMD-related phenotypes in AMD-mimicking cells. As shown in Supplementary Figure S3, only two parameters—Nrf2 and SOD—showed a positive correlation, while 14 parameters showed a negative correlation. The most negatively related factors showed strong negative correlations (−1.0–−0.92) with the α-iso-cubebene concentration, regardless of the function of the factor. These results indicate that increasing α-iso-cubebene concentration was negatively correlated with most AMD-related parameters, whereas positive correlations were observed for Nrf2 and SOD.

## 4. Discussion

Overexposure to high-energy light induces photooxidative stress in the retina, as evidenced by increased superoxide anion generation and pathological changes, including enhanced photoreceptor outer-segment phagocytosis [43,44,45]. Excessive production of ROS in response to BL exposure is a major contributor to AMD [46]. Accordingly, enhancing cellular antioxidant capacity has emerged as a promising therapeutic strategy for AMD-like retinal injury. In this context, plant-derived antioxidants (phytochemicals) have attracted considerable attention as potential AMD therapeutics, owing to their potent biological activities and relatively low toxicity at therapeutic concentrations. As part of our effort to identify a novel single compound with high antioxidant activity from natural products, we investigated the protective effects of α-iso-cubebene isolated from *Schisandra chinensis* against BL-induced damage in A2E-laden ARPE-19 cells and BALB/c mice, established in in vitro and in vivo models of AMD-like retinal injury. In the present study, α-iso-cubebene improved the survival of ARPE-19 cells exposed to AMD-associated stress conditions. This protective activity was accompanied by reductions in oxidative damage and inflammatory signaling, together with preservation of endogenous antioxidant responses. Furthermore, retinal structural alterations and inflammatory responses induced by BL exposure were alleviated following α-iso-cubebene administration in vivo.

To date, numerous studies have demonstrated that natural free radical–scavenging compounds can alleviate blue light–induced retinal damage [47,48]. Among these compounds, tetrahydrocurcumin—a major metabolite of curcumin—was reported to exert protective effects against AMD by activating the Nrf2 pathway and enhancing SOD activity in retinal pigment epithelial cells, thereby reducing intracellular ROS accumulation and oxidative stress [48]. Similarly, saffron-derived components, including crocins, crocetin, and picrocrocin, exhibited potent antioxidant activity and suppressed oxidative stress through the P2X7-mediated calcium signaling pathway in 661W retinal photoreceptor cells [47]. In addition, *Centella asiatica* extract was shown to inhibit A2E oxidation in ARPE-19 cells via activation of the Nrf2/heme oxygenase-1 (HO-1) pathway and preserved outer nuclear layer thickness in a murine model of retinal degeneration induced by N-methyl-N-nitrosourea [49]. In agreement with these previous observations, α-iso-cubebene displayed potent free radical–scavenging activity in our experimental system, and effectively suppressed intracellular ROS accumulation within the concentration range of 40–80 µM. These effects may be mediated by direct free radical scavenging and enhancement of en-dogenous antioxidant defenses potentially involving Nrf2-related antioxidant signaling. Taken as a whole, the current data support the possibility that α-iso-cubebene may be associated with a reduction in oxidative retinal injury by simultaneously modulating several antioxidant and cytoprotective pathways. Although the precise molecular mechanism remains unclear, it is conceivable that α-iso-cubebene may influence the Keap1–Nrf2 regulatory axis, as reported for several antioxidant phytochemicals. However, further mechanistic studies are required to determine whether Nrf2 stabilization and nuclear translocation contribute to the observed increase in antioxidant defenses and protection against oxidative retinal injury [50,51].

Beyond oxidative damage, the progression of AMD is closely associated with abnormal angiogenesis, particularly in neovascular (wet) AMD [52]. Excessive VEGF signaling promotes the formation of immature and leaky blood vessels, which can ultimately lead to central vision loss [53]. Therefore, inhibition of VEGF and MMP expression represents a critical therapeutic strategy for preventing AMD progression [54]. Several natural products have been reported to exert anti-angiogenic effects in retinal cells [55,56]. Among these studies, extracts from *Prunella vulgaris* suppressed the expression of VEGF-A and MMP2 in ARPE-19 cells exposed to A2E and BL [55]. Similarly, red wine extract inhibited VEGF-A secretion and reduced the expression of VEGF receptors in ARPE-19 cells [56]. In our study, the effects of α-iso-cubebene on angiogenesis-related markers were evaluated in ARPE-19 cells exhibiting AMD-like retinal injury to find a novel single compound for AMD. α-Iso-cubebene significantly downregulated the expression of VEGF, MMP2, and MMP9 in A2E- and BL-treated ARPE-19 cells. The overall response pattern observed in the present study was comparable to that reported for several natural products with anti-angiogenic activity. Although additional functional studies are required, the present findings provide preliminary evidence supporting the potential utility of α-iso-cubebene in modulating angiogenesis-related molecular responses.

Inflammation within retinal tissues, particularly in association with neovascularization, plays a critical role in accelerating AMD progression. Therefore, anti-inflammatory agents, in addition to anti-angiogenic therapies, have been proposed as effective treatments for AMD management [57]. Various natural compounds have been reported to exert protective effects by modulating inflammatory signaling pathways in retinal cells. The flavonoid antioxidant baicalin suppressed the expression of inflammasome components, including NLRP3, ASC, and Cas-1, in amyloid β–damaged ARPE-19 cells, and reduced the production of pro-inflammatory cytokines such as IL-1β and IL-18 [58]. Similarly, the plant-derived quinone celastrol inhibited the mRNA expression of IL-6, IL-8, and MCP-1 and attenuated NF-κB signaling by suppressing p65 phosphorylation in lipopolysaccharide-stimulated ARPE-19 cells [59]. In addition, cynaroside decreased the expression of inflammatory cytokines, including IL-1β, IL-18, and TNF-α, and inhibited the activation of inflammasome components in A2E- and BL-treated ARPE-19 cells [60]. Cynaroside also suppressed NF-κB nuclear translocation by inhibiting IKK-β activation and preventing IκB-α degradation, thereby limiting cytokine gene transcription [61]. Consistent with these findings, our results showed that α-iso-cubebene was associated with reduced the protein expression levels of COX-2, iNOS, NLRP3, ASC, and Cas-1, as well as the mRNA levels of *TNF-α*, *IL-1β*, *IL-6*, and *NF-κB*. These observations indicate that α-iso-cubebene effectively attenuates inflammatory responses in retinal cells by affecting the expression of multiple inflammasome-related markers and NF-κB-related inflammatory response.

Meanwhile, the structural disorganization of the retina is widely used as a reliable hallmark in AMD because it directly reflects the cumulative effects of oxidative stress, inflammation, and photoreceptor degeneration on retinal integrity [61]. In particular, thinning of the ONL, disruption of photoreceptor outer segments (OS), and morphological alterations in RPE correlate closely with visual dysfunction and progression of AMD [62,63]. To date, these structural changes in the retina have been significantly improved or preserved by a few single compounds. Resveratrol remarkably inhibited the thinning of light-induced ONL in BALB/c mice, while quercetin significantly protected against retinal degeneration and helped maintain ONL thickness in visual light–exposed BALB/c mice [22,23]. Also, in P23H-rhodopsin transgenic rats, curcumin treatment increased ONL and INL thickness, as well as maintaining photoreceptor outer-segment structure [24]. Some sigma receptor ligands, such as (+)-pentazocine and CM398, protected against photoreceptor loss and RPE damage in AMD-related Abca4^−/−^Rdh8^−/−^ mice [64]. As part of a study to identify a novel single compound capable of protecting retinal tissue from degeneration, we analyzed retinal structural alterations in BL-exposed BALB/c mice following α-iso-cubebene injection. Although direct comparisons should be interpreted with caution because of differences in experimental models and treatment protocols, the degree of retinal structural preservation observed following α-iso-cubebene treatment appears to be broadly consistent with the protective effects reported for other natural compounds—including resveratrol, quercetin, and curcumin—in similar retinal degeneration models [22,24]. In particular, α-iso-cubebene effectively preserved the thickness of multiple retinal layers and attenuated retinal disorganization, supporting its potential efficacy within the range of responses previously reported for established antioxidant compounds. Taken together, these observations expand the current knowledge regarding the biological activity of α-iso-cubebene and support its potential value as a naturally derived compound capable of preserving retinal integrity during AMD progression.

## 5. Conclusions

**Figure 8:**
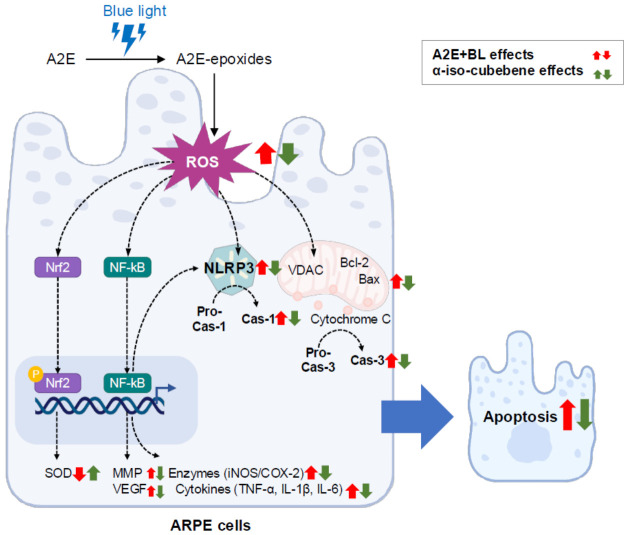
Proposed mechanism for AMD prevention of α-iso-cubebene in A2E+BL-treated ARPE-19 cells. In this scheme, ROS promotes the activation and nuclear translocation of Nrf2 and activates NF-κB signaling, therefore stimulating the secretion of inflammatory cytokines (red arrow indicates BL+A2E induced effects). With the pretreatment with α-iso-cubebene, reduced ROS downregulates NF-*κ*B signaling and translation of inflammatory proteins, which can result in decreased apoptosis (green arrow indicates the prevention effects of α-iso-cubebene). Abbreviations: AMD, age-related macular degeneration; ROS, reactive oxygen species; COX-2, cyclooxygenase-2; iNOS, inducible nitric oxide synthase; BL, blue light; A2E, N-retinylidene-N-retinylethanolamine; NLRP3, NLR family pyrin domain containing 3; Cas-1, caspase-1; TNF-*α*, tumor necrosis factor-*α*; IL-6, interleukin-6; IL-1*β*, interleukin-1*β*; NF-*κ*B, nuclear factor kappa-light-chain-enhancer of activated B cells; MMP, matrix metalloproteinase; Cas-3, caspase-3; Bax, Bcl-2-associated X protein; Bcl-2, B-cell lymphoma-2; VEGF, vascular endothelial growth factor; VDAC, voltage-dependent anion channel; SOD, superoxide dismutase. Red upward arrows indicate upregulation, whereas green downward arrows indicate downregulation.

In this study, we sought to identify a single compound with strong therapeutic potential for the treatment of AMD. To achieve this, the protective effects and potential molecular response of α-iso-cubebene were analyzed in ARPE-19 cells and BALB/c mice models with AMD-like retinal injury. α-Iso-cubebene showed protective effects in ARPE-19 cells against A2E and BL co-treatment, and was associated with reduced reactive oxygen species accumulation, enhanced cellular antioxidant defenses, and alterations in angiogenic, inflammatory, and apoptotic signaling markers. Furthermore, α-iso-cubebene alleviated retinal structural disorganization and reduced inflammatory cytokine expression in BL-exposed BALB/c mice (Figure 8). Therefore, the present findings suggest that α-iso-cubebene may represent a potential natural product–derived single compound for the prevention and management of AMD. Despite these encouraging findings, the present study should be considered a proof-of-concept investigation and has several limitations. First, only a single dose of α-iso-cubebene and a relatively short evaluation period (24 h) were examined in the animal model. Second, in the in vivo study, a positive control was not included, limiting direct comparisons with established retinal protective agents. Third, pharmacokinetic and pharmacodynamic analyses were not performed, restricting a comprehensive assessment of the biological relevance, tissue exposure, and therapeutic efficacy of α-iso-cubebene under physiological conditions. Therefore, future studies are required to investigate dose–response relationships, long-term efficacy, pharmacokinetic properties, bioavailability, and safety profiles. Such investigations will be essential for further evaluating the therapeutic potential of α-iso-cubebene and facilitating its translation toward clinical applications for AMD and related retinal degenerative disorders.

## Data Availability

The data presented in this study are available from the corresponding author upon reasonable request.

## Supplementary Materials

The following supporting information can be downloaded at: https://www.mdpi.com/article/10.3390/cimb48070669/s1.

## Abbreviations

The following abbreviations are used in this manuscript:

AMD	Age-related macular degeneration
A2E	N-retinylidene-N-retinylethanolamine
ARPE-19	Human retinal pigment epithelial cells
BL	Blue light
ROS	Reactive oxygen species
NO	Nitric oxide
SOD	Superoxide dismutase
Nrf2	Nuclear factor erythroid 2–related factor 2
VEGF	Vascular endothelial growth factor
MMP	Matrix metalloproteinase
COX-2	Cyclooxygenase-2
iNOS	Inducible nitric oxide synthase
NLRP3	NLR family pyrin domain containing 3
ASC	Apoptosis-associated speck-like protein containing a CARD
TNF-α	Tumor necrosis factor-α
IL	Interleukin
NF-κB	Nuclear factor kappa-light-chain-enhancer of activated B cells
DPPH	2,2-diphenyl-1-picrylhydrazyl
DMEM	Dulbecco’s Modified Eagle’s Medium
FBS	Fetal bovine serum
PBS	Phosphate-buffered saline
RT-qPCR	Quantitative real-time polymerase chain reaction
SDS-PAGE	Sodium dodecyl sulfate–polyacrylamide gel electrophoresis
HRP	Horseradish peroxidase
LC-MS	Liquid chromatography–mass spectrometry
H&E	Hematoxylin and eosin
ONL	Outer nuclear layer
OPL	Outer plexiform layer
INL	Inner nuclear layer
OS	Outer segment
IS	Inner segment
SPF	Specific pathogen-free
KFDA	Korean Food and Drug Administration
AAALAC	Association for Assessment and Accreditation of Laboratory Animal Care International
AVMA	American Veterinary Medical Association
