# Supplementary material for: α-Iso-Cubebene Alleviates AMD-like Retinal Injury Through Modulation of Oxidative Stress and Inflammatory Response
Source: Curr Issues Mol Biol. 2026 Jun 29;48(7):669. doi: 10.3390/cimb48070669 (PMC13406941; doi:10.3390/cimb48070669)
Supplement: Supplementary file 1 [file cimb-48-00669-s001.zip › cimb-4348986-supplementary.pdf]

*Supplementary Data*

# $\alpha$-Iso-Cubebene Alleviates AMD-like Retinal Injury Through Activation of Nrf2-Mediated Antioxidant Signaling and Suppression of Inflammation

**Ye Ryeong Kim [1,†], Ayun Seol [1,†], Su Jin Lee [1,†], Ji Eun Kim [1], Hee Jin Song [1], Su Jeong Lim [1], Su Ha Wang [1], Ye Eun Ryu [1], Young Whan Choi [2], Sun Il Choi [3,4,*] and Dae Youn Hwang [1,5,*]****Supplementary Figures**

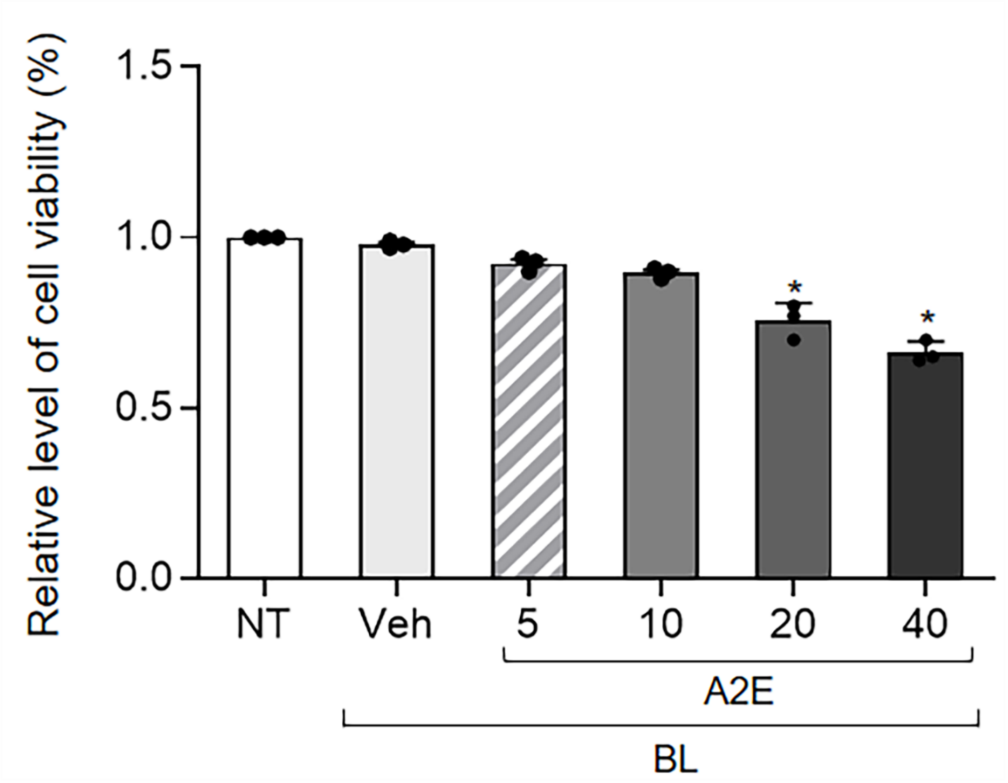


**Figure S1.** Determination of the optimal A2E dosage for cytotoxicity analysis. Cell viability was determined using an MTT assay. Three wells were prepared per treatment group, and the optical density was measured in triplicate. Results are reported as the mean ± SD. * $P < 0.05$ versus the NT group. Abbreviations: NT, Non-treated; Veh, Vehicle (DMSO); VitC, Vitamin C as a positive control; MTT, 3-(4,5-dimethylthiazol-2-yl)-2,5-diphenyltetrazolium bromide; BL, blue light; A2E, bis-retinoid N-retinyl-N-retinylidene ethanolamine.

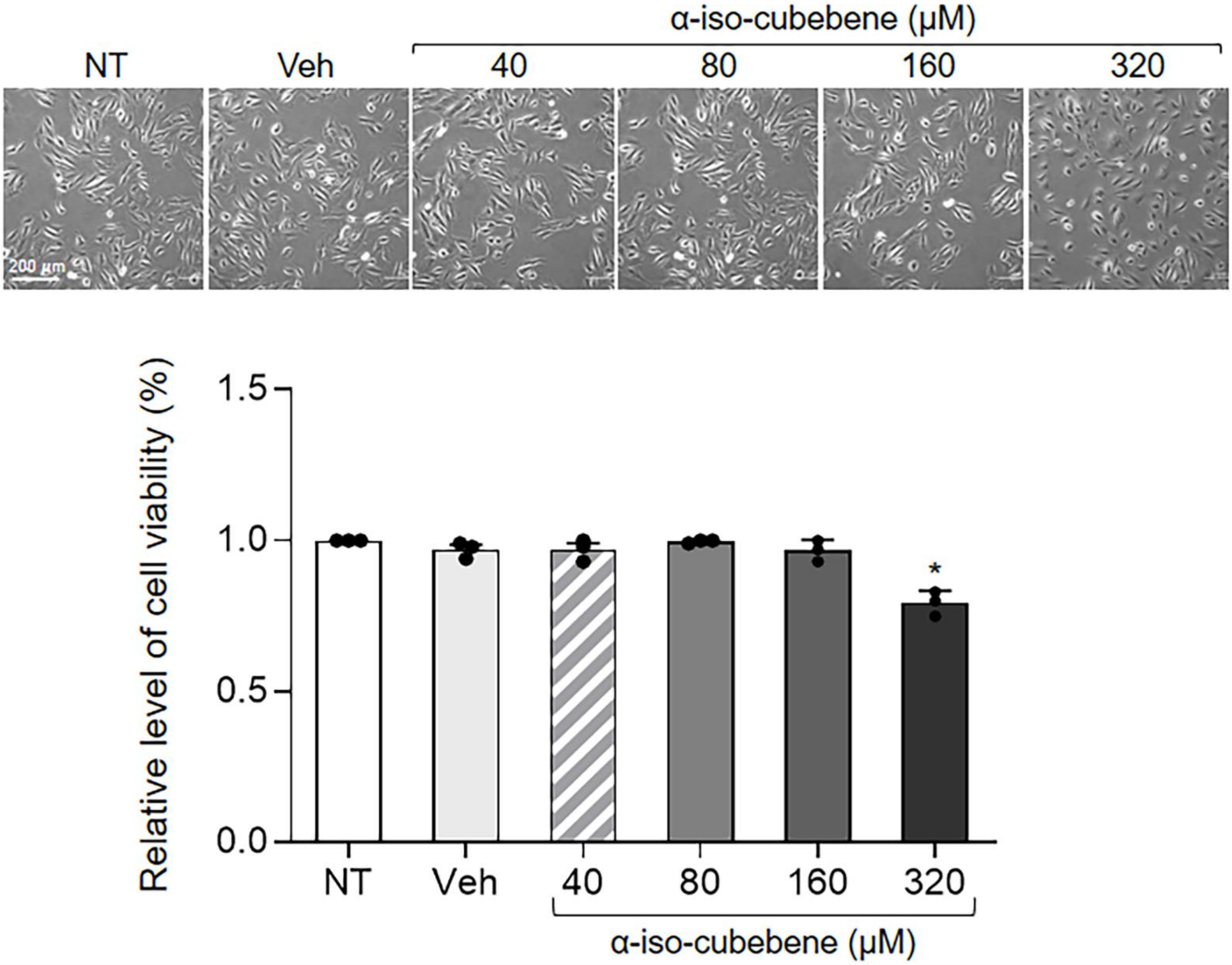


**Figure S2.** Determination of the optimal $\alpha$-iso-cubebene concentration in ARPE-19 cells for anti-AMD effects. Morphological changes were observed under a microscope at 200× magnification. Cell viability was determined using an MTT assay. Three wells were prepared per treatment group, and the optical density was measured in triplicate. Results are reported as the mean ± SD. * $P < 0.05$ versus the NT group. Abbreviations: NT, Non-treated; Veh, Vehicle (DMSO); VitC, Vitamin C as a positive control; MTT, 3-(4,5-dimethylthiazol-2-yl)-2,5-diphenyltetrazolium bromide.

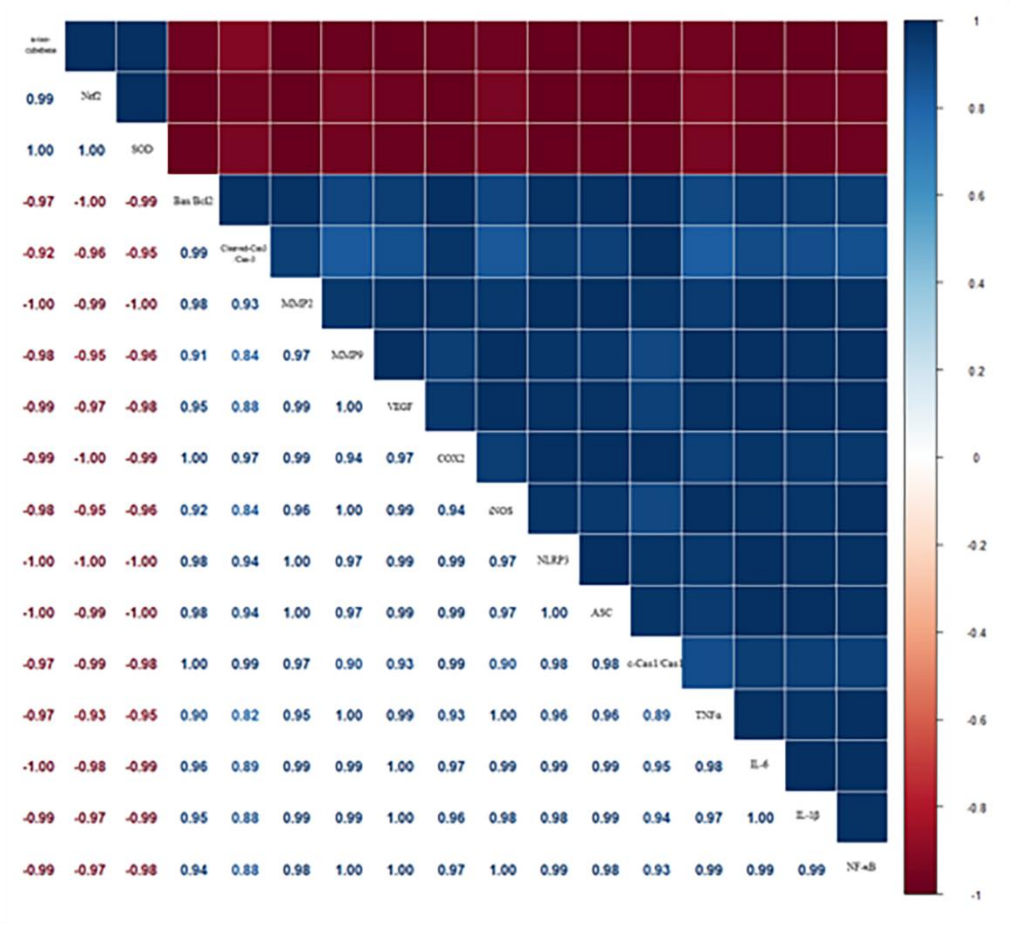


**Figure S3.** Correlogram drawn in Corrplot for the significance of correlative relationship between $\alpha$-iso-cubebene concentration and the measured parameters from ARPE-19 cells. The coloring degree is scaled from strongly positive (dark blue) to strongly negative (dark red). The numbers and colors represent the same values in a symmetrical structure based on the factors located on the diagonal.

**Table S1.** List of antibodies for western blot analyses.

| Name | Host | Company | Dilution |
|---|---|---|---|
| Anti-Nrf2 | Rabbit | Abcam | 1:1000 |
| Anti-SOD | Rabbit | Abcam | 1:1000 |
| Anti-Bax | Rabbit | Cell Signaling Technology Inc. | 1:1000 |
| Anti-Bcl-2 | Rabbit | Invitrogen | 1:1000 |
| Anti-Cas-3 | Rabbit | Cell Signaling Technology Inc. | 1:1000 |
| Anti-MMP2 | Rabbit | Cell Signaling Technology Inc. | 1:1000 |
| Anti-MMP9 | Rabbit | Cell Signaling Technology Inc. | 1:1000 |
| Anti-VEGF | Rabbit | Cell Signaling Technology Inc. | 1:1000 |
| Anti-iNOS | Rabbit | Cell Signaling Technology Inc. | 1:1000 |
| Anti-COX-2 | Rabbit | Cell Signaling Technology Inc. | 1:1000 |
| Anti-ACS | Rabbit | Cell Signaling Technology Inc. | 1:1000 |
| Anti-caspase-1 | Rabbit | Cell Signaling Technology Inc. | 1:1000 |
| Anti-cleaved caspase-1 | Rabbit | Cell Signaling Technology Inc. | 1:1000 |
| Anti-NLRP3 | Rabbit | Cell Signaling Technology Inc. | 1:1000 |
| Anti-β-actin | Rabbit | Sigma-Aldrich | 1:3000 |

**Table S2.** List of primer for RT-qPCR analyses.

| Name | Forward primer sequence (5′-3′) | Reverse primer sequence (5′-3′) |
|---|---|---|
| IL-6 | CCGGGAGCCTCTTGATACAG | AGCCCCTCTCAAAGTCACACA |
| IL-1β | GCAGATTTGCTGGTTGGATT | GGCCATATAGCCTCAAACATGAT |
| TNF-*α* | CTGAGGCCCTCCCACATCT | GGAAAGGAACAAGGCCAACA |
| NF-*κ*B | GTAAC AGCAG GACCC AAGGA | AGCCC CTAAT ACACG CCTCT |
| β-actin | ACGGCCAGGTCATCACTATTG | CAAGAAGGAAGGCTGGAAAAGA |
